# Review of the reverse innovation series in globalization and health – where are we and what else is needed?

**DOI:** 10.1186/s12992-020-00555-6

**Published:** 2020-03-26

**Authors:** Matthew Harris, Viva Dadwal, Shams B. Syed

**Affiliations:** 1 Department of Primary Care and Public Health, Imperial College London, London, UK; 2 Independent Researcher, New York, USA; 3 Associate Faculty Member Department of Health Policy and Management, Johns Hopkins Bloomberg School of Public Health, 624 N. Broadway, Baltimore, MD 21205 USA

**Keywords:** Reverse innovation, Bidirectional learning, Mutual benefit, Reciprocity, International health partnerships

## Abstract

Following advances in industrial strategy and organizational behaviour, as well as post-development debates in international relations, Globalization and Health launched the Reverse Innovation series in 2012, in order to forge an agenda to promote not just the innovativeness of low-income country health systems but to recognize current and advocate for future strengthened knowledge flow between the global south and global north. It was considered to be a timely antidote to a knowledge flow that has traditionally been characterised by unidirectionality of innovation and expertise. Since then, the series provides a repository of research, theory, commentary and debate through which a collective community of practice in Reverse Innovation might emerge and provide an evidence base to promote, support and mainstream this type of knowledge flow. In this Commentary, we review the series as a whole, explore what has been learnt and what needs to come next in terms of empirical research, business models, processes and theoretical contributions to inform reverse innovation.

## Background

Following advances in industrial strategy and organizational behaviour [1], as well as post-development debates in international relations, Globalization and Health launched the Reverse Innovation series in 2012, in order to forge an agenda to promote not just the innovativeness of low-income country health systems but to recognize current and advocate for future strengthened knowledge flow between the global south and global north. The series is different to other series in the journal, such as ‘Innovation in LMICs’ and ‘Health Partnerships’, in that it seeks to support the identification and showcasing of LIC innovation that have subsequently been utilized in HIC. It was considered to be a timely antidote to a knowledge flow that has traditionally been characterised by unidirectionality of innovation and expertise [2, 3]. Globalization brings increasing interconnectedness, it brings improved exposure to innovation from all over the world providing opportunities for improved sharing of ideas and scaling of innovation. Globalization provides opportunities to level the playing field in terms of global health expertise, as well as challenge entrenched perceptions regarding the universality of global health policies. The Reverse Innovation series was launched to provide a repository of research, theory, commentary and debate through which a collective community of practice might emerge focussing on how to promote, support and mainstream this type of knowledge flow.

Since then, there has been some significant contribution to the debate.

First, researchers have contributed examples of high value innovative practice in LICs that HIC health systems would benefit from adopting whether due to their improved effectiveness, access, cost or quality of care. Examples include community engagement strategies [4], cardiovascular protection interventions [5], reflecting on the epistemological roots of social epidemiology [6], novel and effective hospital accreditation systems [7], leveraging families to ensure person centredness in healthcare [8], task-shifting to community health workers at scale [9] and high-value opportunities in frugal surgical innovations [10]. For many, these are paradigmatic shifts in approaches to care, others are simple interventions that the reflexive practitioner would recognise as common sense. All borne from the legacy of ‘doing more with less’, LICs can be repositories of innovation, unconstrained by the highly regulated, highly constrained ecosystems of HICs and the obsession, some would argue, for costly, sophisticated, sustaining innovations that do little to improve access or value, but much to stimulate demand in clinicians.

Second, the series has also provided theoretical contributions examining models of Reverse Innovation, knowledge diffusion and the role of international health partnerships [11–15]. There have been empirical studies of the barriers to Reverse Innovation [16–18] including the use of Implicit Association Tests (IATs) to identify unconscious associations between rich countries and good research, and poor countries and bad research [17]. This threat to scientific universalism has been evidenced in other robustly controlled experiments [18]. Also included have been articles that provide tools to support the identification and translation of innovations from LICs into HICs [19]. Ouma et al [20] provide insights into how Reverse Innovation is experienced from the perspective of the LIC partner, with suggestions for how to develop mutually beneficial partnerships in the long term.

Third, the impact of health partnerships on the HIC volunteer has too been noted. Basu et al. [11] describe the specific learning that Johns Hopkins Armstrong Institute obtained through the health partnerships run with hospitals in Uganda, South Sudan and Liberia. On people-centred care, African colleagues taught them that the family and the care team are one with the patient rather than something that is separate and needs to be reunited with. African partners taught them about doing much with little and how to avoid wasteful practices. Jackson F Doe hospital in Liberia has taught them to get serious over sustainability and to reduce their energy use [13] described their experience of using a Reverse Innovation competition as an opportunity to attract suggestions for reverse innovations and to mobilise Canadian capacity to identify those that have the potential to positively impact health systems.

What have we learnt and where should the series go next? Below, we present five key areas that are ripe for exploration over the next decade.

### Empirical research

Reverse Innovation is about improving dialogue in global health, whereby practical, effective and simple approaches to care developed in low-income countries are explored, trialled and scaled in high-income countries. It is a counterpart to the traditional paradigm of ‘West is Best’. However, reverse innovation is challenging and the lack of empirically grounded examples of reverse innovation in this series suggests that there is still much work to be done in understanding and promoting this type of innovation flow. Of particular importance is documenting well evaluated attempts to reverse innovate and what works at different stages in the innovation pathway. There is a need for studies that explore the challenges around (1) innovation identification; (2) necessary specificities of the adopter site; (3) partnerships and the key success factors to persuade and galvanise support; (4) testing for safety and effectiveness; (5) adaptation strategies to fit local contexts; and (6) economic evaluations to understand where savings can be usefully spread throughout the system. However historical context is key. Indeed, it is challenging to persuade clinicians to learn from LICs that historically have been portrayed as needy, dependent and passive recipients of HIC advice and expertise. So, articles that provide empirical evidence or that theorize on the cognitive, professional and epistemic basis to the challenge of reverse innovation would be important. Its challenging to persuade clinicians to use low-specification or low-cost technologies, even if they do the same or similar job as the highly sophisticated alternatives. So, studies that examine ways to engage clinicians, managers and service users in frugal innovation would be important. Its challenging to persuade clinicians to relinquish responsibilities to a lower cadre or lesser-trained health professional even if these deliver those tasks just as well, if not better. So, studies, even if modelling studies, that provide insights into the costs and potential benefits of training, recruitment and support of lower cadres in HIC would be important.

### Terminology

In the industrial strategy literature, reverse innovation, as a terminology, describes an intra-organizational approach to ideate technologies in contexts with favourable institutional voids in order to create new markets back home [21]. However, the term has been adopted wholesale, and quite uncritically, from this literature. In health care ecosystems, where multiple organizations, such as universities, funders and enterprises may all play a part in reverse innovation alongside governments, its use is a paradox, with connotations that can undermine the very thing it is trying to achieve [16]. So, is there a better term that we should be using that reflects the challenge and the ambition of the process? Suggestions have included ‘co-development’, ‘bidirectional learning’ and ‘mutual reciprocity’ but the field requires thoughtful reviews of the use of language in global health contexts and how these terminologies are applicable in certain settings and not others [22]. The pathway to global innovation flow may indeed be multi-fold and certain terms may resonate more in certain parts of the world than in others.

### Process

There are challenges regarding the definition of reverse innovations and we are not yet clear, despite efforts to create typologies [23] what constitutes a reverse innovation. Innovation journeys are difficult to narrate and trace, but also categorizations of developing and developed countries is itself a challenge. A developing country is not necessarily one that is low-income, or even low-middle-income. Indeed, parts of a low-income country may be fully developed. This blended scenario may be increasing across the world. So what categorisations are really relevant in reverse innovation? A developing country might not have colonial ties with a potential adopter country, so the aspect of reverse innovation that confronts post-development colonial legacies might be irrelevant between some contexts. Does that matter? If so, how? Frugal innovations are not only or even inevitably from low-income countries. So, are the challenges of reverse innovation due to the country-of-origin or the frugal characteristics of the innovation? What is the relative importance of these aspects? The field requires some coherence in this respect and a convergence of terminologies.

### Business models

Reverse innovation is clearly not something that happens either in a vacuum or on its own. It is a managed process involving providers, entrepreneurs, academia and research funders all within a context shaped by national governments. So, what is an appropriate business model to support reverse innovation, given that two types of demand need to be stimulated, the demand for the LIC innovator to access new markets, and demand for the HIC health system to adopt the innovation? Can health systems interested in reverse innovation do so in a way that benefits their patient population, the LIC entrepreneur, the intermediaries that create the synergies and the funders that pump prime the initial demonstration projects? Which organizational forms, such as social enterprises, accountable care organizations, single-payer systems, angel investors, or not-for-profit philanthropy, best serves this process? What is the role of organizations that are focused on global health and human development in facilitating reverse innovation?

### Partnerships

Finally, the focus on the HIC context, around issues of adoption, on creating demand, on adaptation strategies, modelling potential benefits and on advocacy ignore the fact that reverse innovation clearly has two sides. The role of the LIC partner in this is important. How can reverse innovation benefit the LIC partner? How can we ensure that due credit, whether financial or intellectual, is captured by LIC entrepreneurs? Do LIC partners see themselves as in a position to help HIC health systems perform better through low-cost innovation? If not, why not? What power dynamics are in play?

## Conclusion

From a disciplinary perspective, Reverse Innovation cuts across health services research, management science, diffusion of innovation theory, organizational behaviour, cognitive psychology, marketing science and development studies, to name just a few. Therefore, it is unable to be fully accommodated in any one of these academic areas alone. Globalization and Health, with its inter-disciplinary debates focussed on a changing world, is a good home for this series. We call on researchers, policymakers, entrepreneurs, clinicians, development specialists and service users to submit contributions that address the areas we discuss above. Our aim is to advance and develop the field of Reverse Innovation, so that one day, the term becomes obsolete because it no longer matters where the innovation comes from.

## Data Availability

Not applicable.
